# The Gut–Brain Axis in Alzheimer’s Disease: Dietary Influences on Neuroinflammation and Cognitive Decline

**DOI:** 10.1192/j.eurpsy.2026.11266

**Published:** 2026-07-31

**Authors:** H. Masullo

**Affiliations:** Medicine, Unisa, São Paulo, Brazil

## Abstract

**Introduction:**

Alzheimer’s disease (AD), the leading cause of dementia, involves cognitive decline, amyloid and tau pathology, and neuroinflammation. Current treatments offer limited benefit, highlighting the need for preventive strategies. The gut–brain axis links microbiota to brain health, with dysbiosis driving inflammation and decline, while Mediterranean and MIND diets promote microbial diversity and anti-inflammatory protection.

**Objectives:**

To review current evidence on the role of the gut–brain axis in Alzheimer’s disease, with a particular focus on how dietary factors influence gut microbiota, neuroinflammation, and cognitive decline.

**Methods:**

This study is a narrative review. A comprehensive literature search was performed in PubMed/MEDLINE using the keywords Alzheimer’s disease, gut–brain axis, microbiota, diet, neuroinflammation, and cognitive decline.

The search retrieved 59 peer-reviewed articles published between 2022 and 2025, including systematic reviews, narrative reviews, and experimental studies. After screening for relevance, 30 articles were included in the final synthesis.

**Results:**

1. **Gut microbiota dysbiosis and Alzheimer’s pathology**

Patients with Alzheimer’s disease or mild cognitive impairment often show reduced microbial diversity and enrichment of pro-inflammatory taxa, with depletion of short-chain fatty acid (SCFA)-producing bacteria. This imbalance was linked to higher amyloid-β, tau hyperphosphorylation, and microglial overactivation, fostering neuroinflammation and neurodegeneration.

2. **Dietary patterns and microbiota modulation**

Mediterranean and MIND diets were associated with richer microbiota, higher SCFA production, reduced inflammation, and slower cognitive decline. Nutrients such as omega-3 fatty acids, polyphenols, and fibers correlated with preserved hippocampal volume and executive function. Western diets, high in saturated fats and refined sugars, promoted dysbiosis, insulin resistance, and accelerated decline.

3. **Mechanistic insights and interventions**

Preclinical studies showed SCFAs modulate microglia and reduce cytokines. Polyphenol-rich foods attenuated amyloid aggregation and oxidative stress. Small clinical trials with probiotics, prebiotics, and caloric restriction suggested cognitive and inflammatory benefits.

**Conclusions:**

This narrative review underscores the gut–brain axis as central to Alzheimer’s disease. Evidence shows that diet-driven modulation of gut microbiota affects neuroinflammation, amyloid and tau pathology, and cognition. Mediterranean-style diets and nutrient-rich interventions appear protective, while Western diets exacerbate dysbiosis. Targeting the gut–brain axis offers a promising, modifiable approach for prevention and therapy in Alzheimer’s disease.

**Disclosure of Interest:**

None Declared

